# Long noncoding RNAs: a potential novel class of cancer biomarkers

**DOI:** 10.3389/fgene.2015.00145

**Published:** 2015-04-23

**Authors:** Aliaksandr A. Yarmishyn, Igor V. Kurochkin

**Affiliations:** Department of Genome and Gene Expression Data Analysis, Bioinformatics Institute, Agency for Science, Technology and Research, SingaporeSingapore

**Keywords:** long noncoding RNA, biomarkers, cancer

## Abstract

Long noncoding RNAs (lncRNAs) are a novel class of RNA molecules defined as transcripts longer than 200 nucleotides that lack protein coding potential. They constitute a major, but still poorly characterized part of human transcriptome, however, evidence is growing that they are important regulatory molecules involved in various cellular processes. It is becoming increasingly clear that many lncRNAs are deregulated in cancer and some of them can be important drivers of malignant transformation. On the one hand, some lncRNAs can have highly specific expression in particular types of cancer making them a promising tool for diagnosis. The expression of other lncRNAs can correlate with different pathophysiological features of tumor growth and with patient survival, thus making them convenient biomarkers for prognosis. In this review we outline the current state of knowledge about the fast growing field of application of lncRNAs as tumor biomarkers.

## Introduction

The development of high resolution microarray and genome wide sequencing technologies allowed comprehensive characterization of mammalian transcriptomes. The main conclusion from the pioneering in-depth transcriptome studies performed by FANTOM (Functional Annotation Of Mouse genome) and ENCODE (Encyclopedia of DNA Elements) consortia was discovery of the pervasive transcription of the mouse and human genomes with protein coding mRNAs constituting only a minor fraction of the transcribed sequences (Okazaki et al., 2002; Carninci et al., 2005; Birney et al., 2007). The most recent data from ENCODE consortium indicates that around 70% of human genome is transcribed, generating a vast range of noncoding RNAs (Djebali et al., 2012). Based on transcript size noncoding RNAs are classified into small noncoding RNAs (<200 nt) and long noncoding RNAs (lncRNAs; >200 nt). Small noncoding RNAs, particularly miRNAs are well characterized as post-transcriptional regulators of mRNAs and have well established roles in cancer (Garzon et al., 2009; Fabian and Sonenberg, 2012; Kong et al., 2012).

On the other hand, lncRNAs still remain poorly characterized, however, evidence for their importance and functionality is mounting. The number of known lncRNA genes is still rising as the process of their annotation is ongoing. The current version of GENCODE (encyclopedia of genes and gene variants) includes 15,877 human lncRNA genes encoding 26,414 transcripts based on manual curation, computational analysis, and experimental validation (Version 21, June 2014 freeze, GRCh38 – Ensembl 77; Dunham et al., 2012; www.gencodegenes.org).

Long noncoding RNAs share common features with mRNAs, such as many of them are transcribed by RNA polymerase II, undergo splicing, polyadenylation, and 5′-capping (**Table 1**). Similarly, to protein coding genes, lncRNA genes have histone marks of active promoters (H3K4me2/3, H2K9ac, H3K27ac) and actively transcribed gene bodies (H3K36me3; Guttman et al., 2009; Derrien et al., 2012). On the other hand, lncRNAs have several distinct features that distinguish them from protein coding mRNAs (**Table 1**). LncRNAs generally lack or have very little open reading frames (ORFs). Commonly, lncRNA transcripts are shorter and have fewer exons. Unlike mRNAs, which are mostly transported to the cytoplasm, the majority of lncRNAs are localized to the nucleus (Derrien et al., 2012). LncRNAs demonstrate relatively lower expression levels than protein coding genes, however, they exhibit more specific tissue expression patterns (Derrien et al., 2012).

**Table 1:** Comparison of several important features of mRNAs and long noncoding RNAs (lncRNAs) based on GENCODE v7 catalog of human transcripts*.

Feature	mRNAs	lncRNAs
Median transcript length	2453 nt	592 nt
Median number of exons	3	8
Expression levels	Higher	Lower
5’ end supported by CAGE	55%	15%
3’ end supported by polyA signal	51%	39%
Tissue specificity	Lower	Higher
Subcellular localization	Mostly cytoplasmic	Mostly nuclear

*The data shown in **Table 1** are taken from Derrien et al. (2012).

**FIGURE 1:**
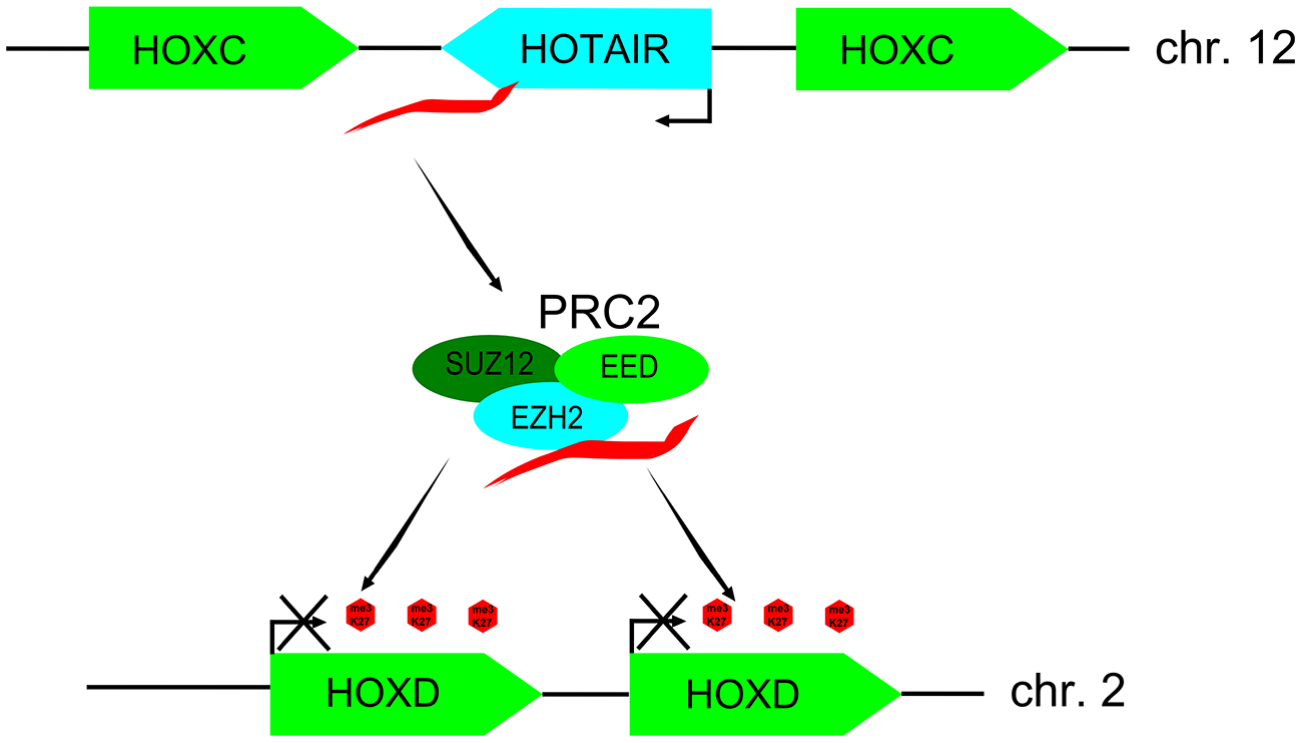
**Model of long noncoding RNA (lncRNA) HOX transcript antisense RNA (HOTAIR) regulating expression of HOX genes in *trans***. LncRNA HOTAIR transcribed from the HOXC cluster of genes (chr. 2) binds PRC2 complex of polycomb-group of proteins and targets it to the HOXD cluster (chr. 12) leading to H3K27 methylation and silencing of neighboring HOXD genes. This figure is adapted from Rinn et al., 2007.

Contrary to the initial view that lncRNAs might be a mere transcriptional noise, several lines of evidence indicate to their functionality (Ponting and Belgard, 2010). Firstly, many lncRNAs demonstrate clear signs of evolutionary conservation and selection (Ponjavic et al., 2007; Guttman et al., 2009). Secondly, expression of many lncRNAs is developmentally and temporally regulated and restricted to certain tissues (Cawley et al., 2004; Ravasi et al., 2006; Mercer et al., 2008). It is becoming increasingly clear that lncRNAs are important regulatory molecules acting at different levels of gene expression, such as chromatin remodeling, transcription, stability, posttranscriptional modifications, and translation. For example, lncRNAs Xist and HOTAIR (HOX transcript antisense RNA) recruit chromatin remodeling complexes, such as Polycomb repressive complex 2 (PRC2) to induce heterochromatin state, thus repressing gene expression at target loci (Zhao et al., 2008; Gupta et al., 2010; **Figure 1**). Indeed, a considerable proportion of lncRNAs has been shown to associate with PRC2 complex, implying that it might be one of the prevalent mechanisms for their functionality (Khalil et al., 2009). LncRNAs directly regulate transcription by different mechanisms, such as via interaction with RNA binding proteins (Wang et al., 2008), repression of promoters (Martianov et al., 2007), acting as co-activators of transcription factors (Feng et al., 2006), or negatively regulating transcription factors by sequestration (Kino et al., 2010; Hung et al., 2011). At the level of pre-mRNA processing, lncRNA MALAT1 can regulate alternative splicing by interacting with several serine/arginine (SR) splicing factors (Tripathi et al., 2010).

Long noncoding RNAs can interact with mRNAs and modulate their translation both positively and negatively. For example, lincRNA-p21 interacts with JUNB and CTNNB1 mRNAs and selectively lowers their translation through a mechanism that includes reduced polysome sizes or ribosome ‘drop-off’ (Yoon et al., 2012). In contrast, lncRNA antisense to Uchl1 gene increases translation of UCHL1 protein in a mechanism dependent on a 5′ overlapping sequence and an embedded inverted SINEB2 element in lncRNA (Carrieri et al., 2012). LncRNA can also regulate stability of target mRNAs through imperfect base pairing between an Alu element in the 3′ UTR of a Staufen 1 (STAU1)-mediated mRNA decay target and another Alu element in a cytoplasmic lncRNA (Gong and Maquat, 2011). This imperfect base-pairing creates a lncRNA–mRNA duplex that binds STAU1 resulting in degradation of mRNA.

With the growing evidence of lncRNA functionality it is of no surprise that they are implicated in diverse pathologic conditions, including cancer. Various lncRNAs have been found to be differentially expressed in cancer and their enforced expression or knock down could result in altered phenotypical responses related to malignant transformation, such as changes in proliferation, invasive potential, or apoptosis. Similarly, to protein coding genes, lncRNAs can be classified into oncogenes and tumor suppressors. This opens opportunities for application of lncRNAs as biomarkers for cancer diagnosis and prognosis. Indeed, the functions of lncRNAs are more likely to correlate with their abundance as they do not encode proteins, making them highly suitable as biomarkers. In this review, we summarize the current knowledge about lncRNAs as potential diagnostic and prognostic biomarkers of cancer.

## Diagnostic Cancer Biomarkers

The tissue specific nature of expression of lncRNAs, which is generally higher than that of protein coding mRNAs (Derrien et al., 2012), makes them potentially advantageous for identification of highly specific diagnostic biomarkers. Whereas some well known lncRNAs, such as HOTAIR are known to be deregulated in a wide spectrum of cancers, several lncRNAs have been described to be highly specific for a particular cancer type.

For example, expression of lncRNA PCA3 (Prostate Cancer Antigen 3) is highly restricted to prostate tissue (Bussemakers et al., 1999). In the same study it was shown by differential display analysis that PCA3 is highly overexpressed in prostatic tumors in comparison with non-neoplastic prostatic tissue of the same patients. Moreover, its expression was not detected in other tumor types or cell lines (Bussemakers et al., 1999). PCGEM1 is another lncRNA gene with highly prostate tissue-specific expression. Interestingly, its elevated expression was associated with high risk groups, such as the African–American population, which is more susceptible to prostate cancer as compared to Caucasian–American or those individuals with family history of prostate cancer as compared to those without family history (Petrovics et al., 2004). Another lncRNA, encoded in 8q24 locus reported to be associated with prostate cancer susceptibility in European and African–American populations, termed PRNCR1 (prostate cancer noncoding RNA 1), was shown to be up-regulated in some of the prostate cancer cells as well as precursor lesion prostatic intraepithelial neoplasia (Chung et al., 2011).

HULC (highly up-regulated in liver cancer) has been identified as highly up-regulated in hepatocellular carcinoma (Panzitt et al., 2007) and colorectal carcinomas that produce liver metastases but not in the primary colon tumors or their non-liver metastases (Matouk et al., 2009). Interestingly, the specific genetic variant rs7763881 in HULC was found to be associated with decreased risk of HCC in persistent carriers of HBV (Liu et al., 2012).

Detection of cancer at the early stages significantly gnosis is the screening for biomarkers by non-invasive methods, such as sampling of extracellular fluids. A fraction of DNA and RNA molecules referred to as circulating nucleic acids (CNAs) are found in blood serum and other extracellular fluids. Changes in the levels of CNAs are associated with tumor burden and malignant progression, thus pointing to their potential as tumor biomarkers easily detected by PCR assays (Schwarzenbach et al., 2011). Several lncRNAs have been characterized as potential biomarkers in human body fluids. The most prominent example of such biomarkers is PCA3, a lncRNA highly expressed is prostate cancer (de Kok et al., 2002). The detection of PCA3 in the urine has been demonstrated to be a more specific marker to diagnose prostate cancer than the commonly used prostate-specific antigen (PSA) and already found wide application in clinics (Hessels et al., 2003; Tinzl et al., 2004; Lee et al., 2011). Similarly, UCA1 (urothelial carcinoma associated 1) transcript detected in urine was shown to be a highly sensitive and specific biomarker of bladder carcinoma (Wang et al., 2006). HULC was detected with high frequency in plasma of patients with hepatocellular carcinoma, making it a promising diagnostic biomarker for this type of cancer (Xie et al., 2013). Several other studies identify promising biomarkers for different types of cancer, such as MALAT1 derived fragment detected in plasma as a biomarker for prostate cancer (Ren et al., 2013), AA174084 found in gastric juice as indicator of gastric cancer (Shao et al., 2014), a set of salivary lncRNAs as potential markers for oral squamous cell carcinoma diagnosis (Tang et al., 2013).

Exosomes are nanovesicles secreted into the extracellular environment from the cells upon fusion of intracellular vesicles with the plasma membrane (Raposo and Stoorvogel, 2013). The molecular content of exosomes replicates that of the releasing cell and reflects its status. The abundance of exosomes in body fluids and possibility to detect tumor specific material of their parental cancer cells makes exosomes a promising tool for non-invasive diagnostics (Properzi et al., 2013). The content of exosomes comprises different kinds of RNA, including lncRNAs (Jenjaroenpun et al., 2013). Indeed, lncRNAs are highly enriched in exosomes compared to donor cells (Batagov et al., 2011; Gezer et al., 2014). For example, lncRNA TUC339 was found to be highly enriched in extracellular vesicles secreted from hepatocellular carcinoma cells where it modulated tumor cell growth and adhesion (Kogure et al., 2013). LincRNA–ROR, another lncRNA from hepatocellular carcinoma derived extracellular vesicles was shown to modulate chemotherapeutic response in this type of cancer (Takahashi et al., 2014). Given the fact that most transcriptome studies aimed to identify biomarkers were focused on miRNAs, shifting the focus to more unbiased characterization that would include other types of transcripts present in cancer derived exosomes would potentially lead to discovery of more lncRNA based biomarkers.

## Prognostic Cancer Biomarkers

Different lncRNAs were shown to be aberrantly expressed in cancer and correlate with tumorigenesis, tumor progression, and metastatic properties in various cancer conditions. Such lncRNA can be involved in both oncogenic and tumor suppressor pathways and their expression can correlate with good or bad prognosis, making them promising prognostic biomarkers (**Table 2**).

**Table 2:** lncRNAs with cancer biomarker potential.

lnc-RNA	Cancer type	Reference	Oncogene Tumor suppressor	Biomarker usability potential
HOTAIR	Breast	Gupta et al. (2010), Bhan et al. (2013), Wang et al. (2013)	Oncogene	Predictor of metastasis and poor survival
	Liver	Geng et al. (2011), Yang et al. (2011), Ishibashi et al. (2013)	Oncogene	Predictor of recurrence and poor survival
	Colorectal	Kogo et al. (2011)	Oncogene	Predictor of liver metastasis and pool survival
	Gastric	Hajjari et al. (2013)	Oncogene	Predictor of lymph node metastasis
	Pancreas	Kim et al. (2013)	Oncogene	Predictor of poor survival
	Lung	Liu et al. (2013a), Nakagawa et al. (2013)	Oncogene	Predictor of metastasis and poor survival
	Esophagus	Lv et al. (2013)	Oncogene	Predictor of metastasis and poor survival
	Cervical	Huang et al. (2014)	Oncogene	Predictor of metastasis and poor survival
MALAT1	Lung adenocarcinoma	Ji et al. (2003), Gutschner et al. (2013)	Oncogene	Predictor of metastasis and poor survival
	Liver	Lai et al. (2012)	Oncogene	Predictor of recurrence after liver transplantation
	Colorectal	Zheng et al. (2014)	Oncogene	Predictor of poor postoperative prognosis
	Cervical	Guo et al. (2010)	Oncogene	
	Bladder	Ying et al. (2012), Han et al. (2013)	Oncogene	Predictor of metastasis
	Uterine	Yamada et al. (2006)	Oncogene	
	Osteosarcoma	Fellenberg et al. (2007)	Oncogene	Predictor of poor response to chemotherapy
H19	Esophagus	Hibi et al. (1996)	Oncogene	
	Breast	Lottin et al. (2002)	Oncogene	
	Lung	Kondo et al. (1995)	Oncogene	
	Bladder	Ariel et al. (2000)	Oncogene	
	Ovarian	Kim et al. (1998)	Oncogene	
	Cervical	Kim et al. (2002)	Oncogene	
	Osteosarcoma	Ulaner et al. (2003)	Oncogene	
	Neck squamous carcinoma	el-Naggar et al. (1999)	Oncogene	
	Liver	Matouk et al. (2007)	Oncogene	
ncRAN	Neuroblastoma	Bown et al. (1999), Yu et al. (2009)	Oncogene	Predictor of poor survival
	Bladder	Zhu et al. (2011)	Oncogene	
	Colorectal	Qi et al. (2014)	Tumor suppressor	Low level predicts poor survival
HULC	Liver	Panzitt et al. (2007), Matouk et al. (2009), Liu et al. (2012)	Oncogene	Diagnostics
GAS5	Breast	Mourtada-Maarabouni et al. (2009)	Tumor suppressor	
	Kidney	Qiao et al. (2013)	Tumor suppressor	
	Pancreas	Lu et al. (2013)	Tumor suppressor	
	Bladder	Liu et al. (2013b)	Tumor suppressor	
	Lung	Shi et al. (2013)	Tumor suppressor	
	Gastric	Sun et al. (2014)	Tumor suppressor	Low level predicts poor prognosis
	Pleural mesothelioma	Renganathan et al. (2014)	Tumor suppressor	Low level predicts poor prognosis
	Liver	Tu et al. (2014)	Tumor suppressor	Low level predicts poor prognosis
PCA3	Prostate	Bussemakers et al. (1999), Hessels et al. (2003)	Oncogene	Non-invasive diagnosis by sampling urine or blood


		Tinzl et al. (2004), Lee et al. (2011)		
UCA1	Bladder	Wang et al. (2006)	Oncogene	Non-invasive diagnosis by sampling urine
PCGEM1	Prostate	Petrovics et al. (2004)	Oncogene	High risk predictor
PRNCR1	Prostate	Chung et al. (2011)	Oncogene	High risk predictor
MEG3	Leukemia	Benetatos et al. (2010)	Tumor suppressor	CpG methylation predicts poor survival
	Pituitary adenoma	Gejman et al. (2008)	Tumor suppressor	
	Meningioma	Zhang et al. (2010)	Tumor suppressor	Loss associated with progression
	Glioma	Wang et al. (2012)	Tumor suppressor	
NBAT-1	Neuroblastoma	Pandey et al. (2014)	Tumor suppressor	Loss associated with progression
LincRNA–ROR	Liver	Takahashi et al. (2014)	Oncogene	Diagnostics by sampling cancer exosomes; indicator of chemoresistance
PCAT1	Prostate	Prensner et al. (2011)	Oncogene	Associated with progression

HOTAIR is one of the most well studied examples of lncRNA implicated in cancer (Wu et al., 2014a). Initially it was identified by Gupta et al. (2010) to be highly overexpressed in primary and metastatic breast cancer tissues as compared to normal breast epithelium. High HOTAIR expression level in primary tumors was shown to be a significant predictor of eventual metastasis and death (Gupta et al., 2010). Enforced expression of HOTAIR in breast carcinoma cells induced genome wide retargeting of PRC2 and, as a result, altered H3K27 methylation and gene expression patterns, increased invasiveness, and metastasis (Gupta et al., 2010). Interestingly, BRCA1, an important suppressor of breast cancer inhibits HOTAIR dependent PRC2 activity by competitive binding to its catalytic subunit EZH2 (Wang et al., 2013). In another breast cancer study it was shown that HOTAIR is regulated by oestradiol in estrogen receptor dependent manner (Bhan et al., 2013).

HOTAIR was also shown to be involved in hepatocellular carcinoma (Geng et al., 2011; Yang et al., 2011; Ishibashi et al., 2013). Overexpression of HOTAIR in tumor tissues was reported to be associated with increased risk of recurrence after hepatectomy, as well as positively correlate with lymph node metastasis (Geng et al., 2011). Similarly, increased HOTAIR was shown to be prognostic factor of tumor recurrence following liver transplantation (Yang et al., 2011). In the same study, knock down of HOTAIR in HCC cell line reduced cell viability and invasion, as well as increased sensitivity to cisplatin and doxorubicin (Yang et al., 2011). Patients with overexpressed HOTAIR had poorer prognoses and larger tumor sizes, more rapid proliferation of tumor cells (Ishibashi et al., 2013).

In colorectal carcinoma high expression of HOTAIR was also shown to correlate with poor prognosis and liver metastasis (Kogo et al., 2011). Positive correlation between expression levels of HOTAIR, the members of PRC2 complex, Suz12, and Ezh2, and H3K27me3 chromatin marks suggests the role of HOTAIR in PRC2 mediated chromatin reprogramming in metastasis (Kogo et al., 2011). Apart from the primary tumors, HOTAIR was also shown to be a negative prognostic factor in the blood of colorectal patients suggesting that measuring the HOTAIR blood levels may provide a minimally invasive test to identify patients with unfavorable prognosis (Svoboda et al., 2014). High expression of HOTAIR associated with advanced stage, lymphatic node metastasis and poor survival was also reported in gastric cancer (Endo et al., 2013; Hajjari et al., 2013; Xu et al., 2013). The increased expression level of HOTAIR positively correlates with Suz12, implying PRC2 dependent mechanism of epigenetic reprogramming in oncogenicity of gastric carcinoma (Hajjari et al., 2013). Also HOTAIR was shown to act as an endogenous sponge of miR-331-3p miRNA, thus abolishing repression of its target HER2, implicated in development of gastric cancer (Liu et al., 2014). In addition to the above described, HOTAIR was reported to be a negative prognosis biomarker for a number of other malignancies, such as pancreatic cancer (Kim et al., 2013), lung cancer (Liu et al., 2013a; Nakagawa et al., 2013), esophageal carcinoma (Lv et al., 2013), cervical cancer (Huang et al., 2014), and colon cancer (Wu et al., 2014b). A meta-analysis study of prognostic capability of HOTAIR in different types of cancer revealed that it is a more reliable predictor of overall survival in patients with digestive system malignancies (Zhang et al., 2014).

Another paradigmal case of lncRNA based cancer biomarkers is MALAT1 (Metastasis-associated Lung Adenocarcinoma Transcript 1). As its name implies, it was discovered as a predictive marker of metastasis development in early stage lung adenocarcinoma (Ji et al., 2003). In a MALAT1 knockout model of human lung cancer cells it was shown that MALAT1 regulates metastatic signature of genes (Gutschner et al., 2013). In such model metastatic capacities of lung adenocarcinoma mouse xenografts were significantly compromised (Gutschner et al., 2013). In the same study, targeting MALAT1 with antisense nucleotides significantly reduced metastasis making it an interesting target for therapy (Gutschner et al., 2013).

In hepatocellular carcinoma (HCC) MALAT1 overexpression is a predictive factor for tumor recurrence following liver transplantation (Lai et al., 2012). The depletion of MALAT1 by siRNA in HepG2 cell line resulted in reduction of cell viability, mobility and invasiveness, as well as increase of sensitivity to apoptosis (Lai et al., 2012). High expression of MALAT1 was shown to be a marker of poor postoperative prognosis in colorectal carcinoma (Zheng et al., 2014). Moreover, the obvious oncogenic role of MALAT1 was demonstrated in a number of other cancer models, including bladder cancer (Ying et al., 2012; Han et al., 2013), cervical cancer (Guo et al., 2010), uterine sarcoma (Yamada et al., 2006), and osteosarcoma (Fellenberg et al., 2007).

H19 is lncRNA expressed from the maternal allele that plays an important role in genomic imprinting during growth and development (Gabory et al., 2010). Loss of imprinting at the H19 locus in paternal allele results in biallelic expression and, therefore, elevated H19 levels in a wide range of cancers (Kondo et al., 1995; Hibi et al., 1996; Kim et al., 1998; el-Naggar et al., 1999; Ariel et al., 2000; Müller et al., 2000; Kim et al., 2002; Lottin et al., 2002; Ulaner et al., 2003). High expression of H19 was shown to be associated with a range of risk factors, such as smoking, exposure to carcinogens and hypoxia (Matouk et al., 2007). Indeed, hypoxia causes up-regulation of H19 in cell lines with mutated p53 (Matouk et al., 2010). H19 is also directly induced by MYC oncogene in different cell lines (Barsyte-Lovejoy et al., 2006). Further adding to the host of oncogenic pathways with the involvement of H19, it serves as a precursor of miR-675, miRNA that down-regulates RB1 tumor suppressor transcript in colorectal cancer (Tsang et al., 2010).

NcRAN (noncoding RNA expressed in aggressive neuroblastoma) is encoded by a gene mapped to chromosome arm 17q, whose amplification is one of the most common genetic abnormalities associated with poor prognosis in neuroblastoma (Bown et al., 1999; Yu et al., 2009). Indeed, the high expression levels of ncRAN were significantly associated with poor prognosis of the neuroblastoma patients (Yu et al., 2009). In addition, ncRAN was found to be upregulated in bladder cancer as compared to normal tissues and confer a set of oncogenic properties, such as increased cell proliferation, migration, and invasion (Zhu et al., 2011). In contrast, down-regulation of ncRAN was shown to be associated with metastatic properties and predict poor overall survival in colorectal carcinoma (Qi et al., 2014).

In addition to lncRNAs with pronounced oncogenic effects, which positively correlate with poor prognosis, a few lncRNAs have been characterized to act as tumor suppressors. One such example is GAS5 (growth arrest-specific 5), a tumor suppressor lncRNA which reduces growth, metabolism, and sensitizes cells to apoptosis by inhibiting glucocorticoid receptor (Kino et al., 2010). GAS5 is down-regulated in a number of cancers, such as breast cancer (Mourtada-Maarabouni et al., 2009), renal cell carcinoma (Qiao et al., 2013), pancreatic cancer (Lu et al., 2013), bladder cancer (Liu et al., 2013b), non-small-cell lung cancer (Shi et al., 2013), gastric cancer (Sun et al., 2014), malignant pleural mesothelioma (Renganathan et al., 2014), hepatocellular carcinoma (Tu et al., 2014). Low level of GAS5 indicates a poor prognosis in cancer patients (Sun et al., 2014; Tu et al., 2014).

Another example of a tumor suppressor lncRNA is lincRNA-p21. This lncRNA was shown to be directly induced by p53 and play a crucial role in p53 mediated transcriptional response (Huarte et al., 2010). Maternally expressed gene 3 (MEG3) is another prominent tumor suppressor lncRNA that acts by increasing p53 activity on specific transcription targets (Zhou et al., 2007). Besides, MEG3 is able to inhibit cell proliferation in the absence of p53 suggesting that this lncRNA may act also via p53-independent pathway (Zhou et al., 2007). Consistently, MEG3 is down-regulated in a number of cancers, including myeloid leukemia (Benetatos et al., 2010), pituitary adenoma (Gejman et al., 2008), meningioma (Zhang et al., 2010), and glioma (Wang et al., 2012).

In conclusion, lncRNAs may act both as oncogenes and tumor suppressors without obvious prevalence of one class over another. In this respect lncRNAs behave similarly, to protein-coding transcripts.

## Prognostic Expression Signatures Based on lncRNAs

Characterizing tumor transcriptomes at the system’s level allows identification of molecular subtypes of cancer and development of predictive and prognostic gene expression signatures. Microarrays have been widely used for the past two decades in preclinical research to characterize tumor gene expression profiles on a genome-wide scale. Most of these signatures were based on protein coding genes as little was known about lncRNAs and their important roles in cancer. However, the widely used commercial microarray platforms in addition to protein coding mRNAs were designed to detect numerous ESTs and non-annotated RNAs. Many of these transcripts are now annotated as *bona fide* lncRNAs, therefore the vast collection of microarray datasets accumulated during the past two decades provides a valuable resource for identification of novel gene expression signatures and biomarkers based on lncRNAs. In our recent study we annotated probe sets detecting lncRNAs on widely used Affymetrix U133 series of microarray platforms by using two approaches: firstly by matching probe sets to Gencode database of lncRNAs and secondly by developing a stringent *in silico* protein coding potential prediction filter (Yarmishyn et al., 2014). Affymetrix U133 microarrays have been found to contain probe sets for the measurement of 1581 lncRNAs (Yarmishyn et al., 2014). Using this resource and previously deposited microarray-based data on expression profiling of neuroblastic tumors we identified 159 lncRNA signature that discriminates between localized and metastatic stages of neuroblastoma, as well as between relapsing and non-relapsing primary tumors (Yarmishyn et al., 2014). The data mining of Affymetrix U133 Plus datasets was also applied to identify lncRNA signatures associated with different histological subtypes and malignancy grades of glioma (Zhang et al., 2012). In the follow up study, the six-lncRNA prognostic signature of glioma significantly associated with overall survival was identified (Zhang et al., 2013). In another study the classification of glioma into three molecular subtypes based on lncRNA expression profiles was proposed (Li et al., 2014). Similarly, using lncRNA mining approach, Hu et al. (2014) identified six lncRNA prognostic signature significantly associated with disease free survival in colorectal carcinoma. LncRNA-based expression profiles were also utilized to classify colorectal cancer samples into five distinct molecular subtypes, each characterized by distinct biological pathways and clinical outcome (Chen et al., 2014).

As high throughput sequencing technology becomes cheaper and more accessible we might expect the influx of *de novo* data from transcriptome sequencing and identification of new biomarkers and diagnostic signatures. For example, Prensner et al. (2011) utilized RNA-Seq approach to identify 121 lncRNAs associated with prostate cancer progression in a cohort of 102 patients. One of these lncRNAs PCAT-1 was further characterized as a regulator of proliferation in prostate cancer cells (Prensner et al., 2011). In a similar study the sequencing of transcriptomes of high and low risk neuroblastoma identified a set of differentially expressed lncRNAs. One of them NBAT-1 was characterized as a tumor suppressor, whose loss contributes to aggressive neuroblastoma by increasing proliferation and impairing differentiation (Pandey et al., 2014). RNA-Seq analysis was also employed by White et al. (2014) to detect lncRNAs associated with lung cancer. In this study the transcriptome sequencing data from a cohort of 567 patients were used to identify 111 novel lncRNAs differentially regulated between tumor and adjacent normal tissue samples. Further meta-analysis revealed that a subset of these lncRNAs was deregulated in a broad range of tumors and another subset was highly deregulated specifically in lung cancers, making the latter a promising source of biomarkers (White et al., 2014).

## Concluding Remarks

The last decade witnessed a vast expansion of knowledge regarding lncRNAs and their important roles in regulation of various biological processes and development of disease. A number of lncRNAs, such as HOTAIR, MALAT and H19 were found to be aberrantly expressed in a number of cancers and extensively characterized as important players affecting the hallmark events of carcinogenesis, such as proliferation, apoptosis, and metastasis. These examples demonstrate that lncRNAs, on par with protein coding genes and miRNAs, have a great potential to be used as cancer biomarkers. Indeed, since lncRNAs are implicated in cancer biology at the level of RNA unlike mRNAs, which represent an intermediate on the way to functioning protein, their levels of expression are more likely to correlate with cancer phenotypes. Also, since proteins and lncRNAs represent different regulatory realms, their combination might increase each other’s power as diagnostic and prognostic tools. It should be noted that the few examples described in this review are just a tip of the iceberg, as the field of lncRNAs is currently evolving and the work of annotation and characterization of lncRNAs is ongoing. With the deep sequencing technology becoming cheaper and more accessible one might expect an explosive growth of newly identified lncRNAs differentially expressed in cancers and associated with various clinical parameters.

## Conflict of Interest Statement

The authors declare that the research was conducted in the absence of any commercial or financial relationships that could be construed as a potential conflict of interest.
